# The Role of Gut Bacteria and Fungi in Alcohol-Associated Liver Disease

**DOI:** 10.3389/fmed.2022.840752

**Published:** 2022-03-03

**Authors:** Liuying Chen, Yixin Zhu, Xiaohua Hou, Ling Yang, Huikuan Chu

**Affiliations:** ^1^Division of Gastroenterology, Union Hospital, Tongji Medical College, Huazhong University of Science and Technology, Wuhan, China; ^2^Department of Medicine, University of California, San Diego, San Diego, CA, United States

**Keywords:** gut dysbiosis, fungi, alcohol-associated liver disease, gut-liver axis, intestinal barrier

## Abstract

Cirrhosis and liver cancer caused by alcohol-associated liver disease (ALD) are serious threats to people's health. In addition to hepatic cell apoptosis and liver inflammation caused by oxidative stress during alcohol metabolism, intestinal microbiota disorders are also involved in the onset and development of ALD. Ethanol and its' oxidative and non-oxidative metabolites, together with dysbiosis-caused-inflammation, destroys the intestinal barrier. Changes of several microbial metabolites, such as bile acids, short-chain fatty acids, and amino acid, are closely associated with gut dysbiosis in ALD. The alcohol-caused dysbiosis can further influence intestinal barrier-related proteins, such as mucin2, bile acid-related receptors, and aryl hydrocarbon receptor (AhR), and these abnormal changes also participate in the injury of the intestinal barrier and hepatic steatosis. Gut-derived bacteria, fungi, and their toxins, such as lipopolysaccharide (LPS) and β-glucan translocate into the liver through the damaged intestinal barrier and promote the progression of inflammation and fibrosis of ALD. Thus, the prevention of alcohol-induced disruption of intestinal permeability has a beneficial effect on ALD. Currently, multiple therapeutic treatments have been applied to restore the gut microbiota of patients with ALD. Fecal microbial transplantation, probiotics, antibiotics, and many other elements has already shown their ability of restoring the gut microbiota. Targeted approaches, such as using bacteriophages to remove cytolytic *Enterococcus faecalis*, and supplement with *Lactobacillus, Bifidobacterium*, or *boulardii* are also powerful therapeutic options for ALD.

## Introduction

According to the most recent WHO data, the burden of alcohol-associated liver disease (ALD) is growing (1). The natural disease course of ALD ranges from asymptomatic liver steatosis, alcoholic hepatitis to the development of cirrhosis, and liver cancer. Alcohol-mediated reactive oxygen species (ROS) formation and hepatic inflammation are the main pathophysiologies of ALD (2). Recently, the role of the gut–liver axis in ALD development and progression has attracted the attention of researchers. Germ-free mice receiving microbiota from patients with alcohol hepatitis gained more serious liver inflammation and disruption of the intestinal integrity, while the mice receiving microbiota from patients without alcoholic hepatitis could reverse the alcohol-caused liver injuries (3).

Cytotoxic effects of ROS during ethanol metabolism are the pathological basis of ALD, and the injuries are exacerbated by hypoxia, inflammation, and bacterial translocation (4). Studies about the role of intestinal flora in ALD have made breakthroughs in the past few years. On one hand, dysbiosis induces intestinal barrier injury, promotes lipopolysaccharide (LPS) and other pathogen-associated molecular patterns (PAMPs) translocation and aggravates inflammatory damage in the liver (5). On the other hand, dysbiosis related exotoxins, such as cytolysin from *Enterococcus faecalis* (6) and *Candidalysin* from *Candida albicans* (7) directly cause hepatocyte death and liver injury. Among the intestinal microorganism, bacteria and fungi are the most studied in ALD. Therefore, we review the interaction between alcohol associated liver disease and the gut microbiome and/or mycobiome to evaluate the contribution of intestinal dysbiosis to ALD. In addition, therapeutic options to restore the intestinal micro-ecosystem will be discussed.

## Gut-Liver Cross Talk

**Figure 1:**
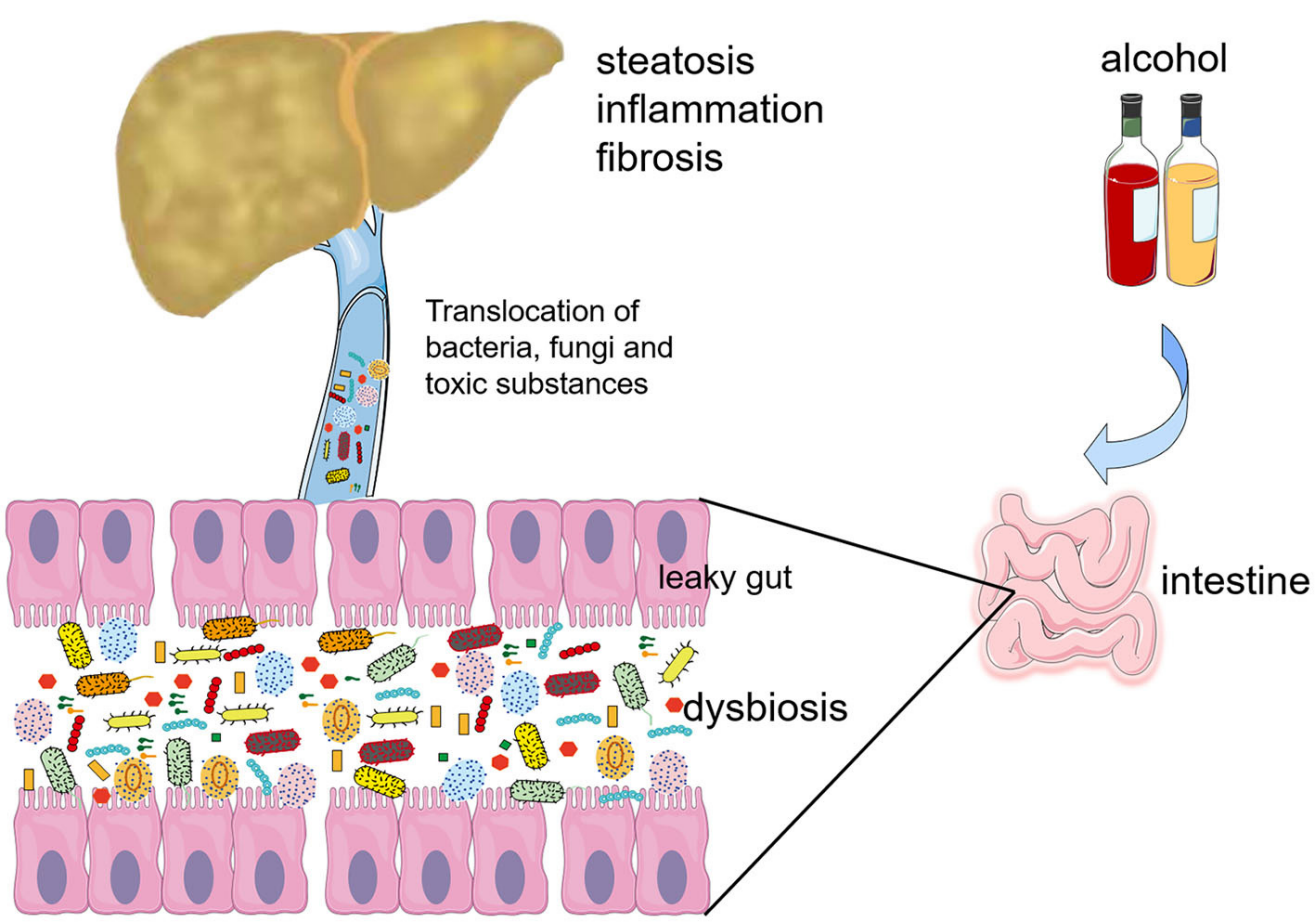
Gut-liver cross talk in alcohol-associated liver disease (ALD). Alcohol induces the gut dysbiosis, mainly manifested as changes in the quantitative and qualitative of intestinal microbiota and fungi. The oxidative and non-oxidative metabolites of ethanol and gut dysbiosis all destroy the intestinal barrier. Gut-derived bacteria, fungi and their toxins, such as lipopolysaccharide (LPS) and β-glucan translocate into the liver though the damaged intestinal barrier and promote the progression of inflammation and fibrosis of ALD.

About 70% of liver's blood supply come from the gut through the portal vein. In addition to bringing nutrients through the portal vein, there is also a chance for intestinal microbiota and their products to enter the liver, especially with increased intestinal permeability. Liver inflammation caused by PAMPs translocated from intestine is the key progression factor of ALD (8) (Figure 1).

### Intestinal Barrier Function

The integrity of the gut barrier plays a key role in prohibiting harmful intestinal materials being translocated into the bloodstream (Figure 1). All patients with various degrees of ALD have disruption of the gut barrier (9, 10). The oxidative and nonoxidative metabolites of ethanol produced by gut bacteria and intestinal epithelial cells disrupt the gut barrier by degrading the tight junction proteins or destroying the interaction of claudin-1 and ZO-1 (11–13). In chronic alcohol feeding mice, enteric dysbiosis-related intestinal inflammatory response causes intestinal barrier injury, and the restoration of eubiosis using non-absorbable antibiotics inhibits intestinal inflammation and barrier dysfunction (14). However, researchers find that mouse colonized with harmful *C. albicans* and cytolytic *E. faecalis* has no effect on intestinal permeability when exposed to alcohol (6, 7). To sum up, the relationship between intestinal dysbiosis and barrier function needs further studies.

The gut vascular barrier (GVB) is another gatekeeper that prevent bacteria translocation across the gut to reach the portal vein. *Salmonella typhimurium* broke the GVB in a manner that is dependent on the declined Wnt/β-catenin signaling in gut vascular endothelial cells (15). In experimental cirrhosis and non-alcoholic fatty liver disease (NAFLD), farnesoid X receptor (FXR) agonists modulate the GVB to reduce bacterial translocation through driving β-catenin activation in endothelial cells (16, 17). Leakage of intestinal vascular endothelia has been observed in experimental ALD mice and patients with ALD (18, 19).

### Gut Microbiota

The intestinal bacteria are significantly altered in patients with ALD and experimental animals. At the phylum level, mice fed with alcohol diet have relatively higher abundances of *Bacteroidetes* and *Verrucomicrobia* when compared to mice fed with a control diet, whereas the mice fed with a control diet have a relative predominance of *Firmicutes* (20). However, alcoholics, with decreased communication of the microbial network in the colon (21) (Table 1), have the lower median abundances of *Bacteroidetes* and the higher median abundances of *Proteobacteria* than the healthy subjects (22). The commensal microbiota is exhausted in ALD patients with and without cirrhosis (23). At the species level, when compared with the healthy group, the numbers of *Bifidobacteria, Lactobacilli*, and *Enterococci* are significantly reduced in the alcoholics (24). Patient with alcoholic cirrhosis have 27 times more *Enterobactericaea* in their feces than healthy volunteers, and *Enterobactericaea* is the most common liver translocated bacterium in patients with cirrhosis (25).

**Table 1:** Changes in intestinal bacteria or fungi and associated metabolites in patients with alcoholic liver disease.

**Groups**	**Different bacteria or fungi**			**Metabolites**	**Reference**
	**Phylum**	**Family**	**Genus/Species**		
Alcoholics with liver disease (*n* = 19) vs. healthy controls (*n* = 18)	*Bacteroidetes*	*Bacteroidaceae*↓			(21)
Alcoholics without liver disease (*n* = 28) vs. healthy controls (*n* = 18)	*Bacteroidetes*	*Bacteroidaceae*↓			
patients with chronic alcohol (*n* = 24) vs. control (*n* = 18)	*Proteobacteria*↑	*Enterobactericaea and Desulfovibrionaceae*↑	*Faecalibacterium* (genus) ↓*Sutterella, Clostridium, and Holdemania* (genus) ↑	Butyric acid(%) of total SCFA concentration↓	(22)
Alcoholism without advanced liver disease (*n* = 72) vs. control (*n* = 60)			*Klebsiella, Lactococcus* (genus) ↑*K. pneumoniae, Lactobacillus salivarius, Citrobacter koseri, Lactococcus lactis subsp. Cremoris* (species) ↑*Akkermansia, Coprococcus, unclassified Clostridiales* (genus) ↓		(23)
Alcoholism with advanced liver disease (*n* = 27) vs. control (*n* = 60)			*Bifidobacterium, Streptococcus Lactobacillus* (genus) ↑*Prevotella, Paraprevotella, Alistipes* (genus) ↓		
Alcoholic patients (*n* = 66) vs. healthy controls (*n* = 24)			*Bifidobacteria, Lactobacilli, Enterococci* (species) ↓		(24)
Alcoholic cirrhotics (*n* = 13) vs. Healthy control (*n* = 7)			*Enterobactericaea, Enterobacter, Bacteroides* (species) ↑		(25)
Alcoholic hepatitis with bilirubin higher than 14.1 mg/dl (*n* = 36) vs. alcoholic hepatitis with bilirubin less or equal 14.1 mg/dl (*n* = 37)			*Veillonella, Enterococcus* (species)↑*Akkermansia* (species) ↓		(26)
Alcoholic hepatitis with MELD higher than 21 (*n* = 54) vs. alcoholic hepatitis with MELD score lower or equal than 21 (n=18)			*unclassified Clostridales, unclassified Prevotellaceae, Anaerostipes* (species) ↓		
Severe alcoholic hepatitis (*n* = 24) vs. healthy controls (*n* = 24)	*Bacteroidetes, Verrucomicrobia* ↓*Fusobacteria*↑*Firmicutes/Bacteroidetes ratio*↑	*Bacilli*↑	*Veillonella* (genus) ↑*Eubacterium*_g23 (genus) ↓		(27)
Bacteria-derived extracellular vesicles (EVs) of severe alcoholic hepatitis (*n* = 24) vs. bacteria-derived EVs of healthy controls (*n* = 24)	*Bacteroidetes, Verrucomicrobia* ↓*Fusobacteria*↑*Firmicutes/Bacteroidetes ratio*↑		*Veillonella* (genus) ↑*Eubacterium*_g23 (genus) ↓		
Alcohol use disorder (*n* = 36) vs. controls (*n* = 36)			*Sutterella, Haemophilus, Staphylococcus, Paraprevotella, Eubacterium, Streptococcus, Odoribacter, Veillonella, Enterococcus, Lactobacillus el at* (genus) ↑*Akkermansia, Blautia, Bifidobacterium, Coprococcus, Dorea, Anaerostipes, Adlercreutzia, Ruminococcus* (genus) ↓		(28)
Alcoholic hepatitis (*n* = 13) vs. control subjects (*n* = 17)				Indole-3-acetic acid, Indole-3-lactic↓	(29)
Active alcohol abuser (*n* = 15) vs. non-alcoholic individuals (*n* = 6)			*Lactobacillus* (species)^*^	Long-chain fatty acids, C15:0 and C17:0^*^	(30)
Alcoholic hepatitis (*n* = 82) vs. controls (*n* = 25)			*Enterococcus faecalis* ↑	Cytolysin ↑	(6)
Alcoholic hepatitis (*n* = 82) vs. alcohol use disorder (*n* = 38)			*Enterococcus faecalis* ↑	Cytolysin ↑	
Alcoholics(*n* = 20) vs. controls (*n* = 8)			*Candida* (genus) ↑*Epicoccum, unclassified fungi, Galactomyces, Debaryomyces* (genus) ↓		(31)
Alcoholic hepatitis (*n* = 91) vs. controls (*n* = 11)			*Candida albicans* ↑	Candidalysin↑	(7)
Alcoholic hepatitis (*n* = 91) vs. alcohol use disorders (*n* = 42)			*Candida albicans* ↑	Candidalysin↑	
Alcohol use disorder (*n* = 15) vs. non-alcoholic controls (*n* = 11)			*Candida* (genus) ↑*Penicillium, Saccharomyces, Debaromyces* (genus)↓		(32)
Alcoholic hepatitis patients (*n* = 59) vs. non-alcoholic controls (*n* = 11)			*Candida* (genus) ↑*Penicillium, Saccharomyces, Debaromyces* (genus) ↓		
Alcohol use disorder (*n* = 66) vs. control subjects (*n* = 18)			*Candida, Debaryomyces, Pichia, Kluyveromyces, Issatchenkia, Scopulariopsis* (genus) ↑*C. albicans, Candida zeylanoides, Issatchenkia orientalis, and Scopulariopsis cordiae* (species) ↑*Aspergillus* (genus) ↓*Kazachstania humilis* (species) ↓		(33)

* *There is significantly correlation between Lactobacilli species and levels of long-chain fatty acids, and their metabolites C15:0 and C17:0 in the fecal samples of active alcohol abusers but not in controls. ↑ means increased, ↓ means decreased*.

The severity of ALD closely relates to the degree of intestinal flora alternations (26). Alcohol dependence is negatively correlated with levels of butyric-producing clostridium species (23). Severe patients with alcoholic hepatitis have increased *Bacilli, Lactobacillales, Veillonella*, and decreased *Eubacterium*_g23, *Oscillibacter* and *Clostridiales* in the fecal compared with the healthy controls (27). The reduced *Akkermansia* and increased *Bacteroides* are used to identify alcohol use disorder patients with an accuracy of 93.4% (28). Cytolysin-positive *E. faecalis* is correlated with the severity of liver disease and mortality in patients with alcoholic hepatitis (34). However, a research from Arun J Sanyal el at. points out that when compared with heavy drinkers, alcoholic hepatitis have more obvious microbiome characteristics, while the bacterial signature between moderate and severe alcoholic hepatitis is not differential (35).

### Gut Fungi

Except for bacteria, gut microorganisms consist of fungi, archaea, and viruses. The role of intestinal fungi in ALD has caught researchers' attention recently. Compared with the control subjects, alcoholic patients have lower fungal species richness and diversity (31). An increased systemic immune response to fungi and their products is associated with increased mortality in patients with alcoholic hepatitis (32). The overgrowth of *Candida*, especially *C. albicans*, is observed in patients with ALD compared with non-alcoholic controls, where *Penicillium* is dominant in the gut of non-alcoholic controls (7, 31, 32). Moreover, the species of *C. albicans* are significantly decreased in alcohol abusers after 2 weeks of abstinence (33). In chronic ethanol diet feeding mice, the commensal fungus *Meyerozyma guilliermondii* is significantly increased compared with mice fed with control diet (36).

## Mechanisms of Intestinal Dysbiosis in the Development Of ALD

**Figure 2:**
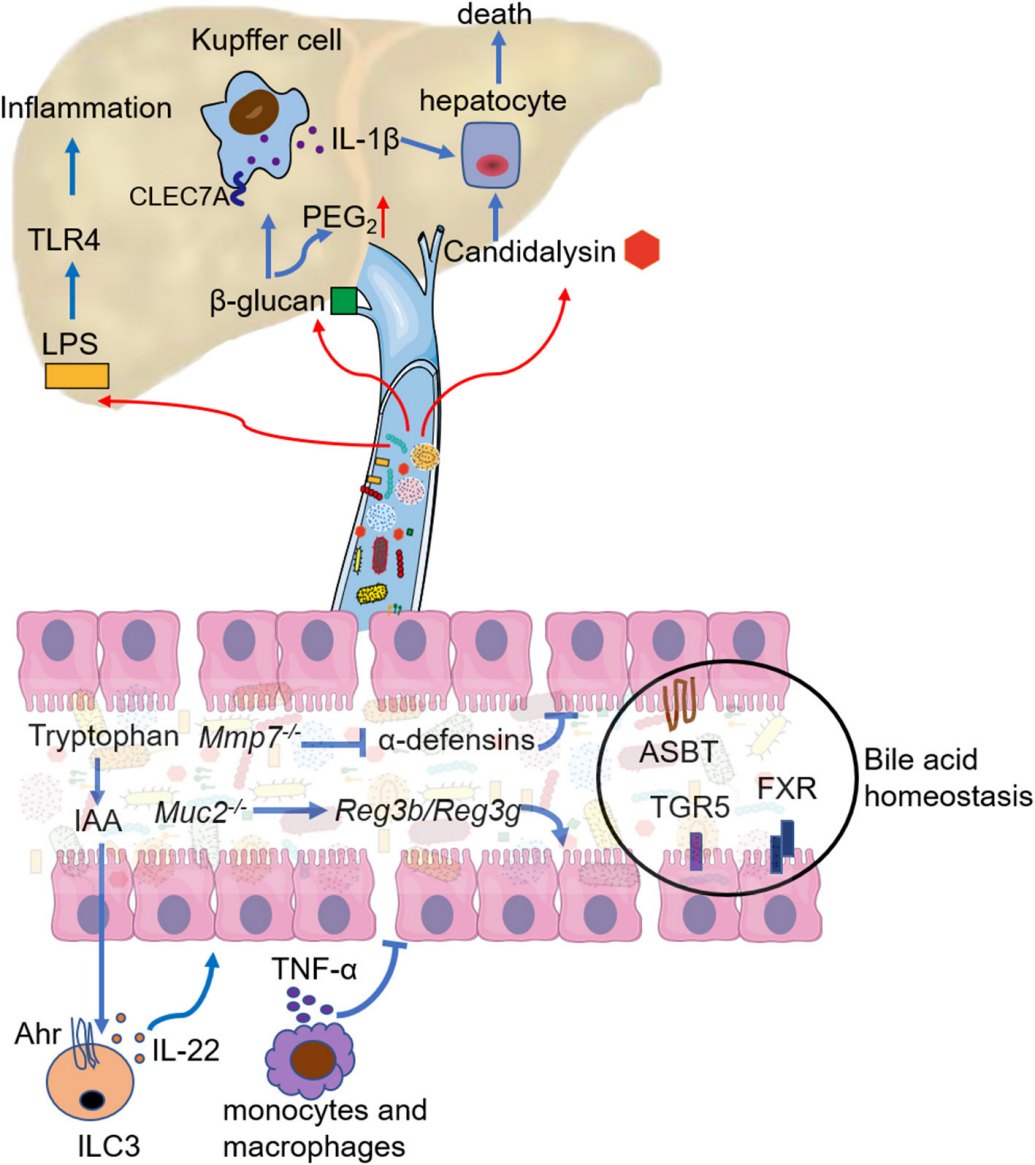
Mechanistic contribution of the gut dysbiosis to ALD. Gut dysbiosis modulates the response of intestinal immune cells, mainlymanifestas decreased IL-22 secretion by innate lymphoid cell 3 (ILC3) and increased TNF-α secretion by intestinal monocytes and macrophages. Both lead to the breakdown of the intestinal barrier function. Deficiency in active α-defensins of intestinal paneth cells (Mmp7 knockout) promotes pathogen associated molecular pattern (PAMP) translocation, but mucin2 deficiency enhances the expression and activity of Reg3b and Reg3g. Bile acid homeostasis is disturbed in ALD, and the regulation of apical sodium-dependent bile salt transporter (ASBT), bile acid receptor (TGR5), and farnesoid X receptor (FXR) could restore bile acid homeostasis and ethanol-associated dysbiosis. LPS from the intestine aggravates liver inflammation mainly through TLR4 signaling. 1,3-β-glucan from the overgrowth of fungi on the one hand binds to the C-type lectin domain family 7 member A (CLEC7A) of Kupffer cells and promotes liver inflammation, on the another hand increases PGE_2_ production in the liver. *Candidalysin* from *Candida albicans* has directly cytotoxic to hepatocytes.

Pathogenic microorganism-related signals and metabolites produced by bacteria or fungi, such as short-chain fatty acids, bile acids, and β-glucan, are involved in the pathology of ALD (37). Restoring intestinal dysbiosis has been found to improve alcoholic liver injury and inflammatory response in patients and experimental mice. We will discuss the mechanism of intestinal dysbiosis in promoting ALD development in the aspects of dysfunction of the intestinal barrier, translocated harmful materials, fatty acid metabolism immunity, bile acid homeostasis, FXR signaling, and AhR signaling (Figure 2).

### Dysfunction of Intestinal Barrier

In addition to the direct effects of alcohol, several molecules have been identified as important factors that contribute greatly to alcohol-related intestinal barrier dysfunction. *Hif1a* knockout in mice intestinal epithelial cells results in a significantly decrease of the *Firmicutes*/*Bacteroidetes* ratio and the *Lactobacillus* level, exacerbating gut leakiness (38). Mucin 2, secreted by goblet cells, protects against pathogens penetrating the inner mucus layer. However, *Muc2* knockout protects mice from alcohol-induced liver injury, prevents the intestinal bacterial overgrowth, and enhances the expression and activity of Reg3b and Reg3g (39). *Mmp7*^−/−^ mouse with a deficiency in active α-defensins of intestinal paneth cells promotes PAMP translocation and worsens the liver damage of alcohol-exposed mice (40).

Immune dysfunction is another important cause of alcohol-related intestinal barrier dysfunction. Intestinal flora is essential for maintaining the immune homeostasis of the gut-liver axis (41). On one hand, the innate and adaptive immune systems influence the component and diversity of intestinal microorganisms (42). On the other hand, gut microbiota has the potential to model intestinal immune responses in healthy and disease states (43). Alcohol feeding significantly upregulates the expression of proinflammatory cytokines interleukin-1 (IL-1) beta, tumor necrosis factor-alpha (TNF-α), interleukin-6 (IL-6), monocyte chemoattractant protein-1 (MCP-1), high mobility group protein box-1 (HMGB-1), interleukin-17 (IL-17), interleukin-23 (IL-23), and inducible nitric oxide synthase (iNOS) in the intestine (44, 45). High TNF-α ruptures the tight junctions by phosphorylating the myosin light chain kinase (MLCK) of intestinal epithelial cells through TNF-receptor I (14). Mice fed with a chronic ethanol diet have decreased the level of fecal immunoglobulin A (IgA) but increased the systemic level of IgA increases compared with the control mice (46). Binge-on-chronic alcohol reduces the number and maturation of mucosa-associated invariant T cells in mice intestines (47). Gut high permeability and dysbiosis result in the highly compromised antibacterial defense of mucosa-associated invariant T cells in patients with ALD (48). Ethanol-induced dysbiosis lower type 3 innate lymphoid cells' production of interleukin-22 (IL-22), and restore IL-22 produced by engineered bacteria in the intestine protecting mice from ethanol-induced steatohepatitis (49). In addition, IL-22 activates the hepatic signal transducer and activator of transcription 3 (STAT3), decreases the hepatic expression of fatty acid transport protein, and ameliorates alcohol related liver inflammatory injury and hepatic oxidative stress (50).

### Translocated Intestinal Bacteria and Their Products

The translocation of intestinal bacteria and their constituent parts, such as peptidoglycan, LPS, flagellin, and CpG DNA, play an important role in ALD progression. Most studies have reported a change in LPS. LPS translocation through the leaky intestine results in circulating endotoxemia and aggravates alcohol-induced liver inflammation through TLR4 signaling in liver (51). Activation of the LPS-TLR4 signaling pathway promotes the release of pro-inflammatory factors, such as TNF-α and IL-6. Hepatocyte TLR4 deficiency decreases lipogenic genes expression, enhances fatty acid oxidation, and reduces inflammatory genes expression of white adipose tissue, then prevents mice from alcohol-induced liver injury (29). Chronic ethanol exposure sensitizes hepatic macrophages to LPS and enhances liver inflammatory damage through TLR4 signaling (52). The absence of MyD88, one of TLR4 signaling adaptor, prevents the progression of hepatic steatosis and inflammation in chronic ethanol-exposed mice (53). However, TLR7-mediated signaling suppresses hepatic injury, steatosis, and inflammation of chronic binge ethanol fed mice (54). In addition, TLR9 signaling protects chronic alcohol exposure mice from hepatic oxidative stress but worsens hepatic inflammation.

Increased exposure to bacterial exotoxins and reduced toxin clearance in the liver also aggravate alcohol-related liver injury and inflammation. Cytolysin from *E. faecalis*, which caused hepatocyte death, are correlated with the severity of liver disease and mortality in patients with alcoholic hepatitis (34). The lack of α1-2-fucosylation in the intestine might contribute to increase the cytolytic *E. faecalis* in chronic ethanol exposed mice (55). Chronic ethanol feeding impairs the hepatic clearance of translocated pathobionts due to the reduced complement receptor of immunoglobulin (CRIg) expression in Kupffer cell (56).

### Fatty Acid Metabolism

Fatty acid metabolism is significantly altered in ALD. Short-chain fatty acids (SCFA), the fermented product of dietary fiber, are the energy source for intestinal epithelial cells and for maintaining barrier integrity (57). The 16S rRNA gene and whole genome shotgun metagenomic analysis have showed the damage of acetyl-coenzyme A (CoA) butyrate synthesizing pathway in butyrate-producing bacterial genera which caused a decreased intestinal level of butyrate by chronic ethanol feeding (58). Tributyrin can inhibit ethanol-induced intestinal barrier and liver injury (59, 60). Saturated long-chain fatty acids are decreased in ethanol intragastric mice, and maintaining the levels of saturated fatty acids in the intestine promote commensal *Lactobacillus* growth and stabilize gut barrier (30). The n3-polyunsaturated fatty acids (PUFAs) can attenuate experimental ALD through decreasing neutrophil chemoattract (61).

### Bile Acid Homeostasis

Bile acid homeostasis is disturbed in ALD. Gut bacteria modulate bile acid metabolism as bile acid diversity is lower and the proportion of taurine-conjugated bile acids is increased in germ-free rats compared with conventional subjects (62). Chronic alcohol administration induces the high expression of choloylglycine hydrolase that is responsible for the deconjugation of bile acid in mice bacteria and the increased levels of unconjugated bile acids, such as cholic acid and muricholic acid in the ileum and plasma, compared with control mice (63). Persons with chronic alcohol consumption have increased bile acid synthesis and bile acid pool in the liver (64). The bile-acid receptor TGR5 maintains biliary homeostasis, as TGR5 deficiency mice have intestinal microbiota dysbiosis, higher plasma and liver levels of secondary bile acids, and greater liver steatosis and inflammation when fed with ethanol diet than WT mice (65). The apical sodium-dependent bile salt transporter (ASBT) inhibitor GSK2330672 attenuates the liver injury of chronic plus binge alcohol mouse by decreasing intestinal bile acid accumulation, and increasing hepatic CYP7A1 expression (66). Restoring bile acid homeostasis by a proliferator-activated receptor-delta agonist seladelpar (MBX-8025) could reduce chronic ethanol-induced liver damage and improve ethanol-associated dysbiosis (67).

### FXR Signaling

The FXR plays an important role in energy metabolism and bile acid synthesis, and thus are extensively studied in ALD. The importance of FXR is different in the gut and the liver in ALD. Intestine-specific FXR knockout mice are more susceptible to ethanol-induced liver steatosis and inflammation compared with WT mice (68, 69). The intestine-restricted FXR agonist fexaramine mitigates hepatic injury and inflammation of chronic ethanol-fed mouse (63). However, FXR deletion in hepatocytes has no effect on the severity of steatosis, inflammation, or liver fibrosis in chronic plus binge alcohol feeding mice, although slight liver lipid deposition and collagen accumulation are increased (70). Nevertheless, an administration of ursodeoxycholic acid (UDCA) attenuates NF-kB activation and inflammatory infiltrations in the liver of FXR knockout mice exposed to ethanol (71). These demonstrate the significance of FXR interaction with gut microbiota in the pathophysiology of ALD.

### Tryptophan and AhR

Aromatic amino acids are important factors to maintain the homeostasis of intestinal flora, among which tryptophan is fully studied in ALD. Tryptophan and tryptophan-derived metabolites are reduced in the fecal and serum of alcoholic hepatitis patients with cirrhosis when compared with nonalcoholic controls, despite that the ability of microorganisms to synthesize tryptophan has improved (72). In ALD animal experiment, *Lactobacillus rhamnosus* GG derived exosome enriched with bacterial metabolites of tryptophan can improve intestinal barrier function through AhR signaling that promotes IL22 production by intestinal immune cells (73). Alcohol-induced intestinal dysbiosis reduces intestinal IL-22 production and indoce-3-acetic acid (IAA) levels. IAA supplementation, as a microbial-derived ligand of AhR, protects mice from ethanol-induced liver steatohepatitis by preventing bacterial translocation to liver (49). Intestinal epithelial cell-specific *Ahr* knockout exacerbates ethanol-induced liver injury through promoting the translocation of *Helicobacter hepaticus* and *Helicobacter ganmani* to liver (74). Ficz (6-formyllindolo (3, 2-B) carbazole), an AhR agonist, reduces ALD liver injury with similar effects to prebiotics. Moreover, alcohol-fed *Ahr* knockout mice eliminates the beneficial effects of prebiotics (75).

### Gut Fungi Relevant Mechanism

The 1,3-β-glucan from the overgrowth of fungi in chronic alcohol-fed mice binds to the C-type lectin domain family 7 member A (CLEC7A) of Kupffer cells and promotes liver inflammation (31). Patients with alcoholic hepatitis have an intense immune response to fungus, as serum anti–*Saccharomyces cerevisiae* antibodies are significantly high compared to patients with alcohol use disorder and nonalcoholic controls (32). *Candidalysin* positive *C. albicans* exacerbate ethanol-induced liver injury not dependent on the further impairment of intestinal barrier function through *Candidalysin*, but dependent on directly cytotoxic to hepatocytes (7). The commensal fungus *M. guilliermondii* induced alcoholic hepatic steatosis is probably due to translocated β-glucan increasing PGE_2_ production in the liver (36).

## Treatments

### Fecal Microbiota Transplantation

Comparing with patients treated with the standard of care, fecal microbiota transplantation from family members attenuates the disease severity and improves the survival rate of severe patients with alcoholic hepatitis (76). In mice models, fecal microbiota transplantation from alcohol-tolerant donor mice to alcohol-sensitive recipient mice can correct alcohol-induced dysbiosis and prevent alcohol-induced liver injury (77).

### Probiotic Bacteria

Many probiotic therapies have been carried out in human patients with ALD and experimental ALD mice where they have received inspiring results. Patients with alcoholic hepatitis who receive 7 days of *Lactobacillus subtilis*/*Streptococcus faecium* have reduced gut-derived microbial LPS and TNF-α level (78). Different species of *Lactobacillus*, such as Lactobacillus plantarum (79), *Lactobacillus acidophilus* (80), *Lactobacillus fermentum* (81), and *L. rhamnosus* GG (82) are all reported to protect against alcohol-induced liver injury through improving intestinal barrier function, modulating gut bacteria, and balancing T_reg_ and T_H_17 cells in peripheral blood of mice. *Akkermansia muciniphila* supplementation decreases ethanol-induced gut leakiness and hepatic injury (83). *L. plantarum* LC27 and *Bifidobacterium longum* LC67 inhibit the activation of NF-κB mediated by LPS, restore the disturbed intestinal flora, and ultimately reduce alcoholic steatosis in mice (84). *Bacillus subtilis* relives alcohol-induced liver damage by reducing bacterial endotoxin translocation and liver inflammation (34). A new strain of *Pediococcus pentosaceus* alleviates ethanol-induced liver injury by increasing the abundance of bacteria that produce SCFAs and strengthening tight junctions of intestinal epithelial cells (85). *Faecalibacterium prausnitzii* and potato starch supplementation attenuate chronic-binge ethanol-induced liver injury by increasing propionate abundance in mice cecum and mitigating the losses of SCFA transporter in the proximal colon (86). VSL#3 treatment prevents intestinal bacteria and their products from spreading to portal circulation and downregulates liver inflammation mediated by TNF-α (87).

### Probiotic Fungi

*Saccharomyces cerevisiae* var. *boulardii* which is anti-carcinogenic, antibacterial antiviral, antioxidant, and able to reduce serum cholesterol level, has been used for treating various gut-related diseases (88). *Saccharomyces boulardii* administration attenuates acute liver injury (89). Besides, *S. boulardii* administration attenuates hepatic steatosis, low-grade inflammation, and changes the gut microbiome (90). *Hanseniaspora osmophila, Lachancea thermotolerans*, and *S. cerevisiae* strains are proved to have the most potential as health-promoting probiotics (91). However, it has also been reported that *Clostridium difficile* colitis and neutropenic patients have S. *cerevisiae* fungemia after treatment with *S. boulardii* as probiotic (92, 93). For future treatments, doctors should concern about the potential risk when prescribing fungal probiotics, especially to immunocompromised patients.

### Prebiotics

Some amino acids, fatty acids, and probiotic fermentation are found to alleviate alcoholic liver disease. Ethanol feeding exhausts protein thiols, raises oxidized protein thiols in mice gut, while glutamine complement can attenuate the protein thiol oxidation of distal colonic mucosa (94). What's more, glutamine prevents the ethanol-induced disruption of the tight junction by EGFR-dependent mechanism (95, 96). L-cysteine attenuates acetaldehyde-induced transepithelial electrical resistance (TEER), and inhibits the ROS injury of Caco-2 cells (11). Tributyrin supplementation has been found to attenuate both acute and chronic-binge ethanol induced intestinal leakage and liver damage (60, 97, 98). For chronic alcohol intragastric mice, supplementation with saturated fatty acids can enhance the intestinal barrier and reduce alcohol-induced liver injury (30). The fermentation broth of the mixture of *Pueraria lobata, Lonicera japonica*, and *Crataegus pinnatifida* by *L. rhamnosus* 217-1 is reported to alleviate alcohol-induced intestinal microbiome disorders, and reduce oxidative stress and inflammatory signals in the liver (99).

### Lifestyle and Medical Intervention

#### Diet Regulation

Dietary inulin and flaxseed oil treatment both attenuate the hepatitis of chronic alcohol exposed mice *via* modulating liver inflammatory response and restoring of the gut microbiota dysbiosis (100, 101). Dietary okra seed oil consumption attenuates lipid metabolic disorder and gut dysbiosis of ALD mice (46).

#### Traditional Medicine

Water-insoluble polysaccharide from *Wolfiporia cocos* reduce liver steatosis caused by chronic ethanol feeding, and suppress the overgrowth of intestinal fungi and *Proteosbacteria* (36). Pomegranate prevents intestinal leakage and liver inflammatory damage caused by alcohol abuse through inhibiting the gut oxidative and nitrative stress (102). Kaempferol alleviates acute alcoholic liver injury in mice by regulating intestinal tight junction protein, butyric acid receptor, and butyric acid transporter expressions (103). Ginkgo biloba compound and puerarin ameliorate experimental alcoholic liver injury by downregulating the expressions of TNF-α, lipopolysaccharide binding protein (LBP), CD14, and TLR4 in liver, and upregulating the expression of tight junction proteins in the intestine (104, 105). The rice bran phenolic extract relieves alcohol caused intestinal microbiota dysbiosis, barrier dysfunction, and liver inflammation (106).

## Conclusions

Gut dysbiosis promotes the development of ALD. The role of fungi in ALD is also important, which deserves further study. As an important barrier for pathogenic microorganisms from the intestine to the portal vein, the mechanism of intestinal blood barrier injury in ALD needs to be clarified. A research recently pointed out chronic alcohol exposure will result in insufficient anti-bacterial immunity of the body, as the number of MAIT cells in peripheral blood of patients with alcohol-related cirrhosis was significantly reduced and their function was impaired (48). Attention should be paid to the high risk of bacteria infection in ALD patients. There is a clear causal link between intestinal translocation PAMPs and liver inflammation (40). Therefore, it is of great significance to further elucidate the mechanism of intestinal barrier injury in ALD. Microecological disorder is an important cause of intestinal barrier impairment, and microbiota-based treatments are the powerful therapeutic options for ALD (107).

## Author Contributions

HC, LY, and XH designed the review and revised the manuscript. LC collected the data and drafted the manuscript. YZ revised the manuscript. All authors have approved the final version.

## Funding

This study was supported by the National Natural Science Foundation of China (No. 82000561 to HC; Nos. 81974078, 81570530, and 81370550 to LY; Nos. 81974062 and 81720108006 to XH), Department of Science and Technology, Hubei Provincial People's Government (No. 2020FCA014 to XH), and the Science foundation of union hospital (No. 2021xhyn005 to HC).

## Conflict of Interest

The authors declare that the research was conducted in the absence of any commercial or financial relationships that could be construed as a potential conflict of interest.

## Publisher's Note

All claims expressed in this article are solely those of the authors and do not necessarily represent those of their affiliated organizations, or those of the publisher, the editors and the reviewers. Any product that may be evaluated in this article, or claim that may be made by its manufacturer, is not guaranteed or endorsed by the publisher.
